# MicroRNA-10a-3p Improves Cartilage Degeneration by Regulating CH25H-CYP7B1-RORα Mediated Cholesterol Metabolism in Knee Osteoarthritis Rats

**DOI:** 10.3389/fphar.2021.690181

**Published:** 2021-06-03

**Authors:** Xiaochen Li, Li Zhang, Xiaoqing Shi, Taiyang Liao, Nongshan Zhang, Yifan Gao, Runlin Xing, Peimin Wang

**Affiliations:** ^1^ Department of Orthopedics, Affiliated Hospital of Nanjing University of Chinese Medicine, Nanjing, China; ^2^ Jiangsu Province Hospital of Chinese Medicine, Nanjing, China; ^3^ Key Laboratory for Metabolic Diseases in Chinese Medicine, First College of Clinical Medicine, Nanjing University of Chinese Medicine, Nanjing, China

**Keywords:** osteoarthritis, cartilage degeneration, miR-10a-3p, cholesterol metabloism, CH25H, CYP7B1, ROR alpha

## Abstract

Osteoarthritis (OA) is a worldwide degenerative joint disease that seriously impaired the quality of life of patients. OA has been established as a disease with metabolic disorder. Cholesterol 25-hydroxylase (CH25H) was proved to play a key role in cartilage cholesterol metabolism. However, the biological function and mechanism of CH25H in OA remains further investigation. Growing researches have proved the vital roles of miRNAs in OA progression. In this study, we screened out miR-10a-3p through high-throughput miRNA sequencing which may bind to CH25H. Molecular mechanism investigation indicated that miR-10a-3p is an upstream target of CH25H. Functional exploration revealed miR-10a-3p suppressed the inflammatory responses, cholesterol metabolism and extracellular matrix (ECM) degradation in primary chondrocytes. Moreover, rescue assays implied that miR-10a-3p reversed CH25H plasmids induced inflammatory cytokine production and ECM degradation. Furthermore, the OA rat model was established to explore the function of miR-10a-3p *in vivo*. The results showed that miR-10a-3p can recover the OA features through targeting CH25H/CYP7B1/RORα axis. In conclusion, these findings implied a crucial role of miR-10a-3p/CH25H/CYP7B1/RORα axis in OA, which may provide a promising therapeutic strategy for OA.

## Introduction

Osteoarthritis (OA) is one of the most disabling joint diseases, characterized by degeneration articular cartilage, synovial inflammation and fibrosis, osteophyte formation and subchondral bone alterations (Hunter and Bierma-Zeinstra, 2019). There are potential causes of osteoarthritis, including inflammation, mechanical damage, oxidative stress, aging, obesity and diabetes. Currently, osteoarthritis is considered to be associated with metabolic disorders (Kulkarni et al., 2021). This perspective arises from the wide association of OA with metabolic syndrome, with higher incidence of this condition in OA patients than in the population without the disease, and a more severe progression of OA in patients with metabolic syndrome (Villalvilla et al., 2020). Therefore, it is necessary to explore the metabolic regulatory mechanism underlying OA pathogenesis which is essential for identifying more therapeutic targets for OA treatment.

Recent studies have proved the role of cholesterol metabolism in the pathogenesis of osteoarthritis, which is involved in the regulation of matrix metalloenzyme in chondrocytes (Choi et al., 2019). Retinoic acid-related orphan receptor α (RORα) was found to mediate the induction of osteoarthritis by alterations in cholesterol metabolism, which acts as downstream receptor of cholesterol hydroxylases, including cholesterol 25-hydroxylase (CH25H) and 25-hydroxycholesterol 7α-hydroxylase (CYP7B1) (He et al., 2021). Osteoarthritic chondrocytes have increased levels of cholesterol because of enhanced uptake, upregulation of CH25H and CYP7B1 and increased production of oxysterol metabolites. However, the upstream regulation mechanism of CH25H remains unknown.

MicroRNAs (miRNAs) are recognized to regulate various diseases progression, including OA, by combining with mRNAs (Duan et al., 2020; Reynard and Barter, 2020; Tavallaee et al., 2020). For instance, miR-520d-5p regulates chondrocyte metabolism by targeting HDAC1 (Lu et al., 2020). MiR-455-3p reduces apoptosis and alleviates degeneration of chondrocyte by targeting PTEN (Wen et al., 2020). Therefore, the miRNA-mRNA interaction plays a pivotal role the progression of OA (Jiang et al., 2020). Thus, miRNAs have the potential to regulate chondrocyte cholesterol metabolism by targeting CH25H.

This study focused on the function and targeted pathways of miRNAs in modulating chondrocyte extracellular matrix (ECM) homeostasis by regulating cholesterol metabolism. Our results may provide a new therapeutic strategy for OA via targeting miR-10a-3p.

## Methods

### Animals

Twenty-four eight-week-old SD male rats were obtained from the Vital River Animal Technology (Beijing, China). Animals were maintained in a specific pathogen-free laminar-flow housing apparatus under controlled temperature, humidity, and 12 h light/dark regimen. The protocol about animal care and usage was approved by the Animal Care and Use Committee of Nanjing University of Chinese Medicine (Approval number: ACU200904) and followed the Guide for the Care and Use of Laboratory Animals of the National Institutes of Health.

### Isolation and Culture of Chondrocytes

The cartilage tissue of rat knee joint was mechanically cut into 1 mm^2^ pieces and digested with DMEM containing 1 mg/ml type II collagenase at 37°C for 6 h. Following cell dissociation, the samples were filtered through a cell strainer. Then, chondrocytes were collected by centrifugation at 1,000 rpm for 8 min and cultured in DMEM supplemented with 10% FBS and antibiotics (100 U/ml penicillin, 100 ug/ml streptomycin). Cells were cultured at 37°C in a humidified 95% air and 5% CO_2_ atmosphere. All the functional experiments were conducted using primary cultured chondrocytes from passages 3–6 (Qi et al., 2021).

Chondrocytes were stimulated with lipopolysaccharide (LPS) (1 μg/ml) for 6 h to stimulate the inflammatory response. The chondrocytes were exposed to complete culture medium with same volume of saline served as control.

### Cell Transfection

MiR-10a-3p mimic, miR-10a-3p inhibitor, miR-10a-3p nagetive control or CH25H-pEX-1 were obtained from Ribobio Company (Ribobio Biotechnology Co., Ltd., Guangzhou, China) and transfected into the chondrocytes using Lipofectamine 2000 (Invitrogen, Unites States) following the protocols provided by the manufacturer. Then, chondrocytes were incubated for 6 h, the culture medium was replaced with complete culture medium, and the cells were incubated for 24 h for further experiments.

### MiRNA Library Construction and Sequencing

MiRNA library preparation and sequencing were conducted by Oebiotech Company (OE Biotech, Inc., Shanghai, China). Briefly, total RNAs were extracted from chondrocytes purified from 2 ml of plasma. Both 3′ and 5′ adaptors were added to each end, respectively, followed by reverse transcription and polymerase chain reaction (PCR) amplification. The PCR products derived from the 18- to 30- nucleotide RNA molecules were purified by electrophoresis and sequenced using the Illumina HiSeq 4000 platform. TargetScan were used to predict target genes of miRNAs. The sequencing data supporting these studies can be found at Sequence Read Archive (PRJNA728870, https://dataview.ncbi.nlm.nih.gov/object/PRJNA728870?reviewer=vuvpn1nuci7rc41i5ufg8d1jj3).

### Dual Luciferase Report

The CH25H 3′-UTR, which contains putative binding sites for miR-10a-3p, was cloned into vectors (Genechem Co., Ltd., Shanghai, China), which contains firefly luciferase as the primary reporter gene and Renilla luciferase as the control reporter gene. The miR-10a-3p binding site in the CH25H 3′-UTR and its mutated version (sequences was shown in Supplementary Material) were cloned into luciferase reporter plasmids. MiR-10a-3p mimics (Genechem Co., Ltd., Shanghai, China) were co-transfected with these reporter plasmids into 293T cells. The cells were harvested 24 h post-transfection, and the luciferase activities were analyzed with the dual-luciferase reporter assay system (Promega, United States), according to the manufacturer’s instructions. The relative values of Firefly luciferase activity were determined by normalizing with Renilla luciferase activity for transfection efficiency.

### Model of ACLT-Induced KOA Procedure

The KOA model of rats was conducted according to the previous literature description. Twenty-four rats were randomly divided into four groups (sham group, KOA group, KOA + miR-10a-3p-NC group, KOA + miR-10a-3p agomir group). The bilateral knees of rats were exposed using an 8–10 mm medial parapatellar approach with the patella laterally dislocated before the anterior cruciate ligament (ACL) was transected in a manner that did not injure the articular cartilage (He et al., 2020; Huang et al., 2021). The skin was then stitched layer by layer. The rats were not restricted in activities and diet after operation. The sham group served as control. Agomir of miR-10a-3p was obtained from Ribobio Biotechnology (Ribobio Biotechnology Co., Ltd., Guangzhou, China) to overexpress miR-10a-3p in rat. Fourteen days after surgery, KOA + miR-10a-3p-NC group and KOA + miR-10a-3p agomir group were injected of agomir or negative control of miR-10a-3p into knee joint. 28 days after injection, rats were sacrificed, and knee joint tissue were collected.

### Histopathological Analysis

For Safranine O and Fast Green staining, cartilage tissues were fixed in 4% paraformaldehyde, soaked in EDTA, embedded in paraffin, and cut into slices. According to previous reports (He et al., 2020), Osteoarthritis Research Society International (OARSI) scoring system was used to evaluate the degeneration of cartilage.

### Western Blot

Cartilage tissues were weighed and then lysed by RIPA lysate on the ice for 15 min. Then, samples were centrifuged with 12,000 g/min for 15 min at 4°C. The supernatant was removed. Cultured chondrocytes were washed and lysed by RIPA. BCA protein assay kit (Beyotime Biotechnology Co., Ltd., Shanghai, China) was used to quantify the protein levels. The protein samples were electrophoresed in SDS-PAGE to separate protein bands. Proteins were transferred from gel onto PVDF membrane, blocked with 5% non-fat dry milk for 2 h. The PVDF membrane was incubated with monoclonal rabbit antibodies specific for MMP-3 (ab52915, Abcam), MMP-13 (MAB13426, Sigma-Aldrich), SOX9 (ab185966, Abcam), Collagen II (ab188570, Abcam), CH25H (sc-293256, SantaCruz), CYP7B1 (ABN182, Sigma-Aldrich) and RORα (ab256799, Abcam) overnight at 4°C. On the next day, membranes were washed and then incubated with second antibody for 2 h. The bands were visualized by ECL method (Beyotime Biotechnology Co., Ltd., Shanghai, China) and the overall gray value of protein bands (average gray value x gray value area) was quantified with Photoshop CS5 software to calculate the target protein relative value (target protein gray value/internal reference overall gray value).

### Quantitative Reverse Transcription-Polymerase Chain Reaction

Total RNA was extracted from cartilage tissues or chondrocytes by using Trizol (Invitrogen, United States). The reverse transcription was performed by using PrimeScript RT Master Mix according to the manufacturer’s instructions. QRT-PCR was performed using SYBR Premix Ex Taq II according to the manufacturer’s instructions on an ABI PRISM 7,500 (Applied Biosystems, United States). The primer sequences are shown in Table1. All primers were obtained from Sangong Biotech (Shanghai, China).

**TABLE 1:** Sequences of primers.

Gene	Forward primer (5′–3′)	Reverse primer (5′–3′)
CH25H	ACG​GAG​CAA​AGT​TGC​AGT​TAA	GGA​GGA​CCA​CTC​AGG​TTA​CGA
CYP7B1	GAA​GTC​CTG​CGT​GAC​GAA​AT	CCT​CAG​AAC​CTC​AAG​AAT​AGC​G
RORα	CTA​CCA​GAA​CAA​GCA​GAG​A	CGAACTCCACCACATACT
MMP3	GAG​GAC​AAA​TTC​TGG​AGA​TTT​GAT​G	GTG​AAG​ATC​CGC​TGA​AGA​AGT​AAA​G
MMP13	CAG​ACA​GCA​AGA​ATA​AAG​AC	CAA​CAT​AAG​CAC​AGT​GTA​AC
SOX9	ACTTGCACAACGCCGAG	CTG​GTA​CTT​GTA​ATC​CGG​GTG
COL2α1	TCC​TAA​GGG​TGC​CAA​TGG​TGA	GGA​CCA​ACT​TTG​CCT​TGA​GGA​C
Aggrecan	TCC​GCT​GGT​CTG​ATG​GAC​AC	CCA​GAT​CAT​CAC​TAC​GCA​GTC​CTC
Adamts4	GGT​GGC​AGA​TGA​CAA​GAT​G	AGT​CGT​TCG​GAG​GGT​TTA​G
Adamts5	GCA​TCA​TCG​GCT​CAA​AGC​TAC​A	TCA​GGG​ATC​CTC​ACA​ACG​TCA​G
GAPDH	TGA​CCT​CAA​CTA​CAT​GGT​CTA​CA	CTT​CCC​ATT​CTC​GGC​CTT​G
miR-10a-3p	CGC​GCA​AAT​TCG​TAT​CTA​GG	AGT​GCA​GGG​TCC​GAG​GTA​TT
miR-10a-3p RT	GTC​GTA​TCC​AGT​GCA​GGG​TCC​GAG​GTA​TTC​GCA​CTG​GAT​ACG​ACT​ATT​CC	
U6	CTCGCTTCGGCAGCACA	AAC​GCT​TCA​CGA​ATT​TGC​GT
U6 RT	GTC​GTA​TCC​AGT​GCA​GGG​TCC​GAG​GTA​TTC​GCA​CTG​GAT​ACG​ACA​AAA​TA	

### Levels of Total Cholesterol, Free Cholesterol and Cholesterol Ester

Total cholesterol, free cholesterol and cholesterol ester in cartilage and chondrocytes supernatant were tested according to the manufacturer’s instructions (Solarbio Science & Technology Co., Ltd., Beijing, China).

### Enzyme-Linked Immunosorbent Assay

IL-1β and TNF-α in serum and chondrocyte supernatant were detected according to the manufacturer’s instructions (Jinyibai Biological Technology Co., Ltd., Nanjing, China).

### Statistical Analysis

All the experiments were performed independently three times (*n* = 3). Data are presented as mean ± SD. Differences among multiple groups were determined using one-way analysis of variance followed by Tukey’s post-hoc test, with *p*-values < 0.05 considered significant.

## Results

### CH25H/CYP7B1/RORα Is Upregulated in Osteoarthritis Cartilage

**FIGURE 1:**
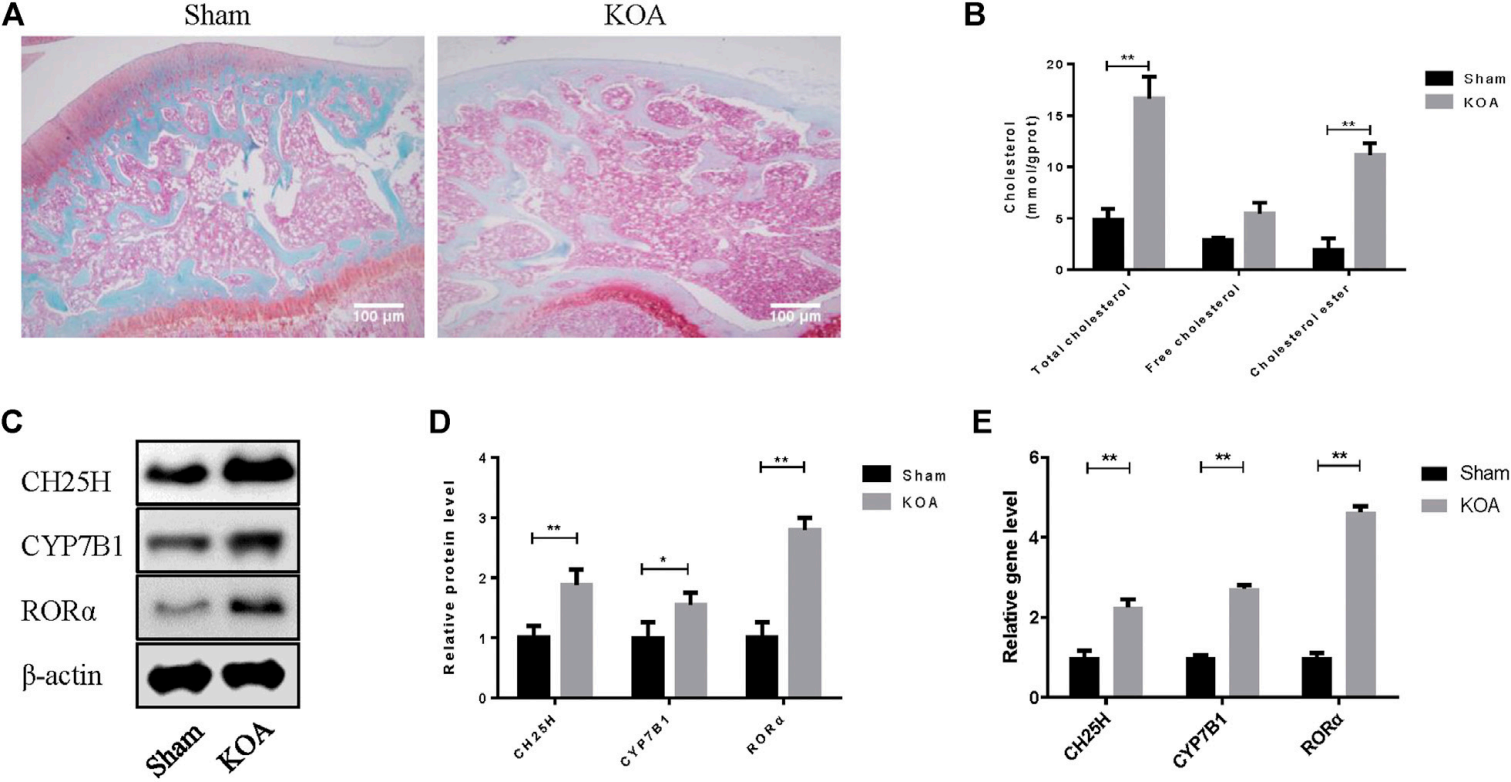
CH25H is involved in cholesterol metabolism disorders in KOA. **(A)** Saffron Red & Fast green staining of cartilage tissue, 100×, Scale bar = 100 μm. **(B)** Total cholesterol, free cholesterol and cholesterol ester were detected in cartilage. **(C**,**D)** CH25H, CYP7B1, and RORα expression in catilage were analyzed via western blotting. The band intensity was quantified by normalizing to β-actin (*n* = 3). **(E)** CH25H and CYP7B1 expression in cartilage were analyzed via qRT-PCR. Quantitative data were presented as mean ± SD. *p < 0.05, **p < 0.01.

Safranine O staining was first conducted to observe the architecture and pathological features of OA joint. OA cartilage shows more superficial fibrillation and loss of collagen and proteoglycan compared with sham group (Figure 1A). Then, the level of CH25H and CYP7B1 in cartilage of OA rats were detected by western blot and qRT-PCR analysis. As shown in Figures 1B,C, both protein and mRNA level of CH25H and CYP7B1 were significantly higher in cartilage from OA group than that from sham group. These results indicated that CH25H/CYP7B1 may contribute to OA progression in OA rats which is consistent with previous researches^3^. However, the upstream regulatory gene that targeted CH25H in OA cartilage remains unknown and it needs to be further explored.

### MiRNAs Profiling Between Normal and Lipopolysaccharide-Stimulated Chondrocytes

**FIGURE 2:**
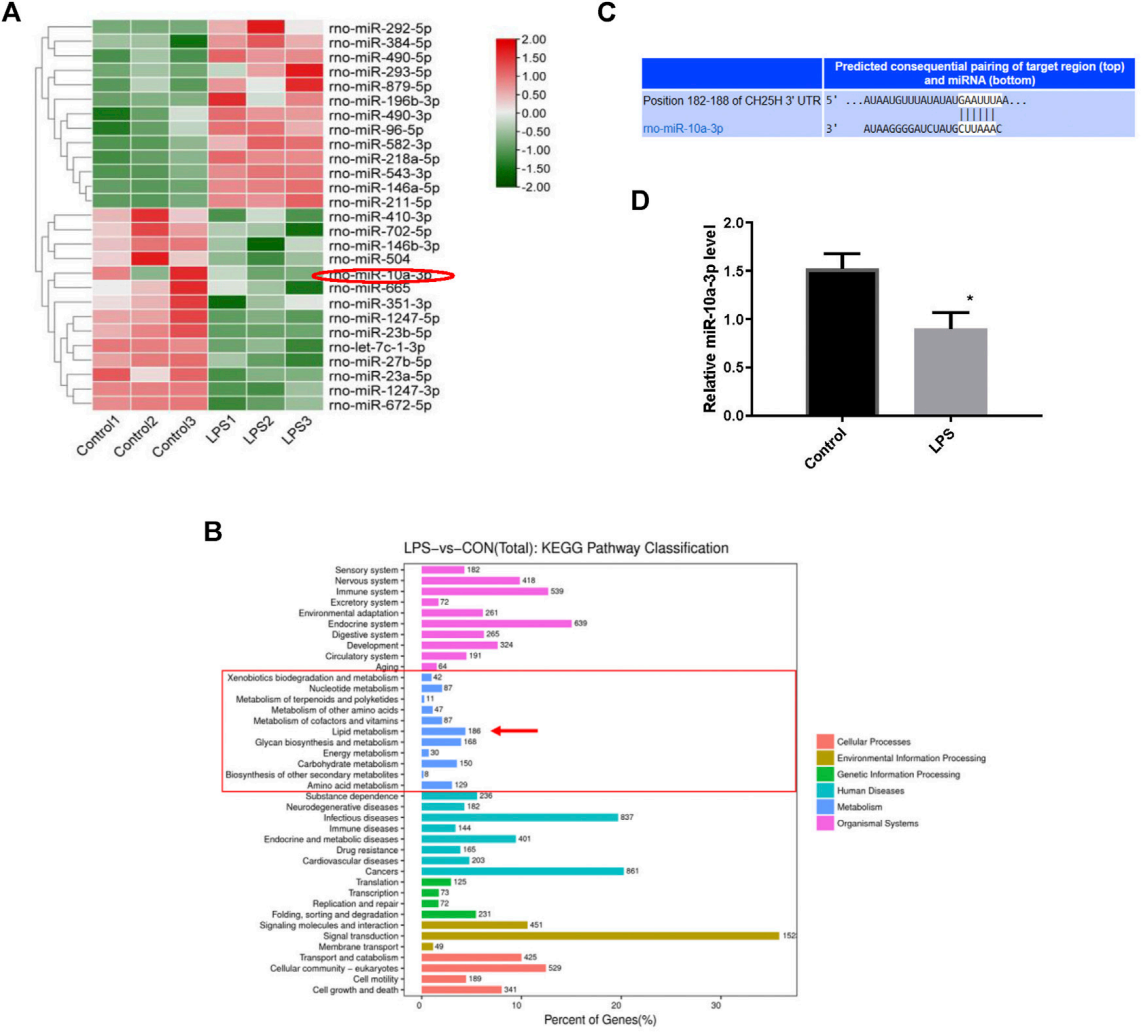
MiRNAs profiling between normal and LPS-stimulated chondrocytes. **(A)** Differential expression levels of miRNAs between the Control and LPS-stimulated chondrocytes. **(B)** The putative binding site between miR-10a-3p and CH25H mRNA was predicted by TargetScan. **(C)** Different expression of miRNA target gene enrichment pathways from KEGG analysis. **(D)** Levels of miR-10a-3p expression in control and LPS-stimulated chondrocytes, as determined by qRT-PCR analysis (*n* = 3). The quantitative data are presented as the mean ± SD. *p < 0.05 vs. control.

To further probe the underlying molecular mechanism of cholesterol metabolism disorders in OA cartilage, we investigated chondrocytes miRNA profiles using high-throughput miRNA sequencing. A total of 27 specific mature miRNAs were obtained from the miRNA sequence results using the screening criteria of *p* < 0.05 for the *t*-test (Figure 2A). Lipid metabolism related miRNAs were highly enriched according to KEGG pathway analysis (Figure 2B). CH25H has been proved to regulate cartilage cholesterol metabolism in OA as previously reported. Importantly, we found that miR-10a-3p is the only miRNA predicted to bind to CH25H among all mature miRNAs with significant changes (*p* < 0.05) by using an online software Targetscan tool (http://www.targetscan.org/vert_72/) (Figure 2C and Supplementary Material). Next, we performed qRT-PCR to validate the high throughput sequencing results. The results showed that LPS stimulation could downregulate miR-10a-3p compared with control chondrocytes (Figure 2D).

### MiR-10a-3p Can Bind with the 3′UTR of CH25H

**FIGURE 3:**
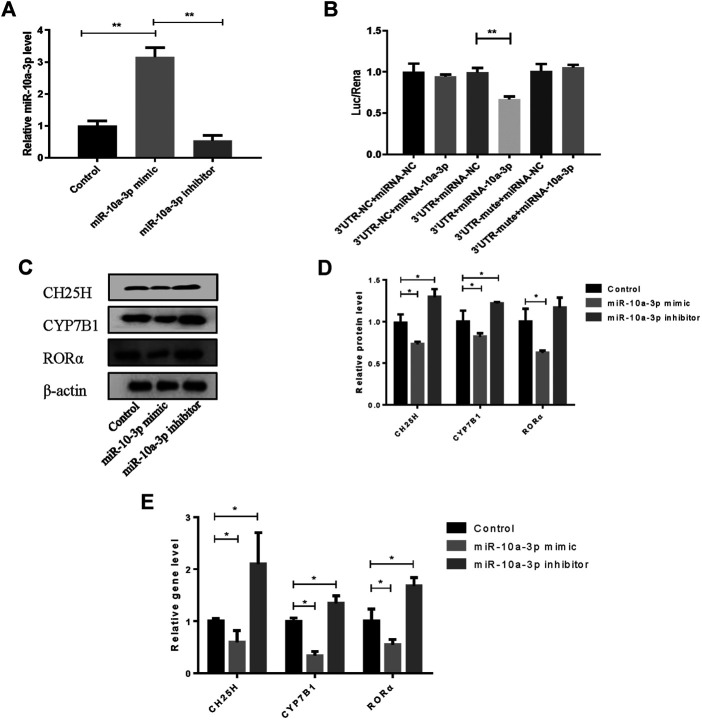
MiR-10a-3p can bind with the 3′UTR of CH25H. **(A)** Transfection efficiency of miR-10-3p inhibitor and mimic were detected by qRT-PCR (*n* = 3). The quantitative data are presented as the mean ± SD. **(B)** The relative luciferase activity was measured after HEK-293T cells were co-transfected with the wild type or mutant 3′UTR of CH25H luciferase reporter and the miR-10-3p negative control or mimic (*n* = 3). **(C**,**D)** CH25H, CYP7B1 and RORα expression in chondrocytes were analyzed via western blotting. The band intensity was quantified by normalizing to β-actin (*n* = 3). **(E)** CH25H and CYP7B1 expression in chondrocytes were analyzed via qRT-PCR. Quantitative data were presented as mean ± SD. *p < 0.05, **p < 0.01.

The overexpression efficiency of miR-10a-3p mimic or knockdown efficiency of miR-10a-3p inhibitor were detected by qRT-PCR analysis in OA rat chondrocytes. The results showed that transfection of miR-10a-3p mimic increased miR-10a-3p expression significantly which was decreased by transfection of miR-10a-3p inhibitor (Figure 3A). As described above, miR-10-3p could bind to 3′UTR of CH25H according to predicted binding sites from Targetscan. To further validate the interaction between miR-10a-3p and CH25H, luciferase reporter assay was conducted. The luciferase activity was significantly decreased in HEK-293T cells by co-transfected with wild type CH25H 3′UTR and miR-10a-3p mimic. The miR-10a-3p mimic caused decreased luciferase activity was impaired by co-transfecting with mutant CH25H 3′UTR. There was no prominent change in luciferase activity of mutant CH25H 3′UTR transfection (Figure 3B). Then, western blot and qRT-PCR analysis indicated that miR-10a-3p mimic suppressed CH25H, CYP7B1 and RORα in both protein and mRNA level which was enhanced by miR-10a-3p inhibitor in LPS-stimulated chondrocytes (Figures 3C,D). Hence, miR-10a-3p was chosen for further exploration.

### MiR-10a-3p Participates in Cholesterol Metabolism Disorders via CH25H/CYP7B1/RORα Axis in Chondrocytes

**FIGURE 4:**
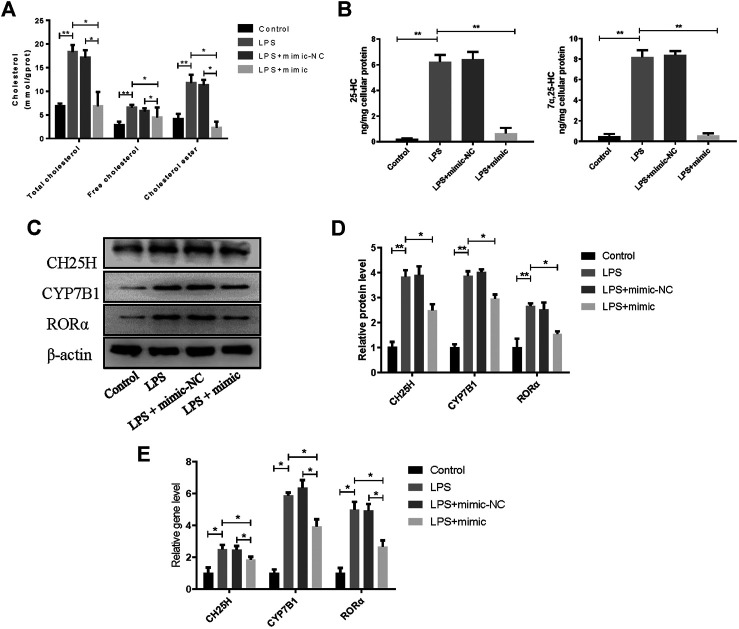
MiR-10a-3p participates in cholesterol metabolism disorders via CH25H/CYP7B1/RORα axis in chondrocytes. **(A)** Total cholesterol, free cholesterol and cholesterol ester were detected in chondrocytes. **(B)** 25-HC and 7α, 25-HC were detected in different group of chondrocytes. **(C)** CH25H, CYP7B1, and RORα expression in chondrocytes were analyzed via western blotting. The band intensity was quantified by normalizing to β-actin (*n* = 3). **(E)** CH25H, CYP7B1, and RORα expression in chondrocytes were analyzed via qRT-PCR. Quantitative data were presented as mean ± SD. *p < 0.05, **P < 0.01.

To confirm the role of miR-10a-3p in chondrocytes, miR-10a-3p/CH25H axis on cholesterol metabolism disorders was investigated by using mimic of miR-10a-3p. As previous researches reported, CH25H/CYP7B1/RORα axis regulates cholesterol metabolism in chondrocytes. Therefore, total cholesterol, free cholesterol and cholesterol ester in chondrocytes were detected. Compared with control group, LPS stimulation induced significantly enhanced levels of total cholesterol, free cholesterol and cholesterol ester, while miR-10a-3p mimic administration reversed the enhanced concentration (Figure 4A). CH25H converts cholesterol to 25-hydroxycholesterol (25-HC) and CYP7B1 metabolizes 25-HC to 7α, 25-HC in chondrocytes. As showed in Figure 4B, LPS stimulation resulted in increased production of 25-HC and 7α, 25-HC and miR-10a-3p mimic could significantly reduce the production of 25-HC and 7α, 25-HC. Then, western blot and qRT-PCR confirmed the increase of CH25H, CYP7B1, and RORα in both protein and mRNA level caused by LPS stimulation (Figures 4C,D). With the administration of miR-10a-3p mimic, activation of CH25H/CYP7B1/RORα could be repressed in LPS-stimulated chondrocytes.

### MiR-10a-3p Regulates Inflammation and Extracellular Matrix Homeostasis in LPS-Stimulated Chondrocytes via Targeting CH25H/CYP7B1/RORα Axis

**FIGURE 5:**
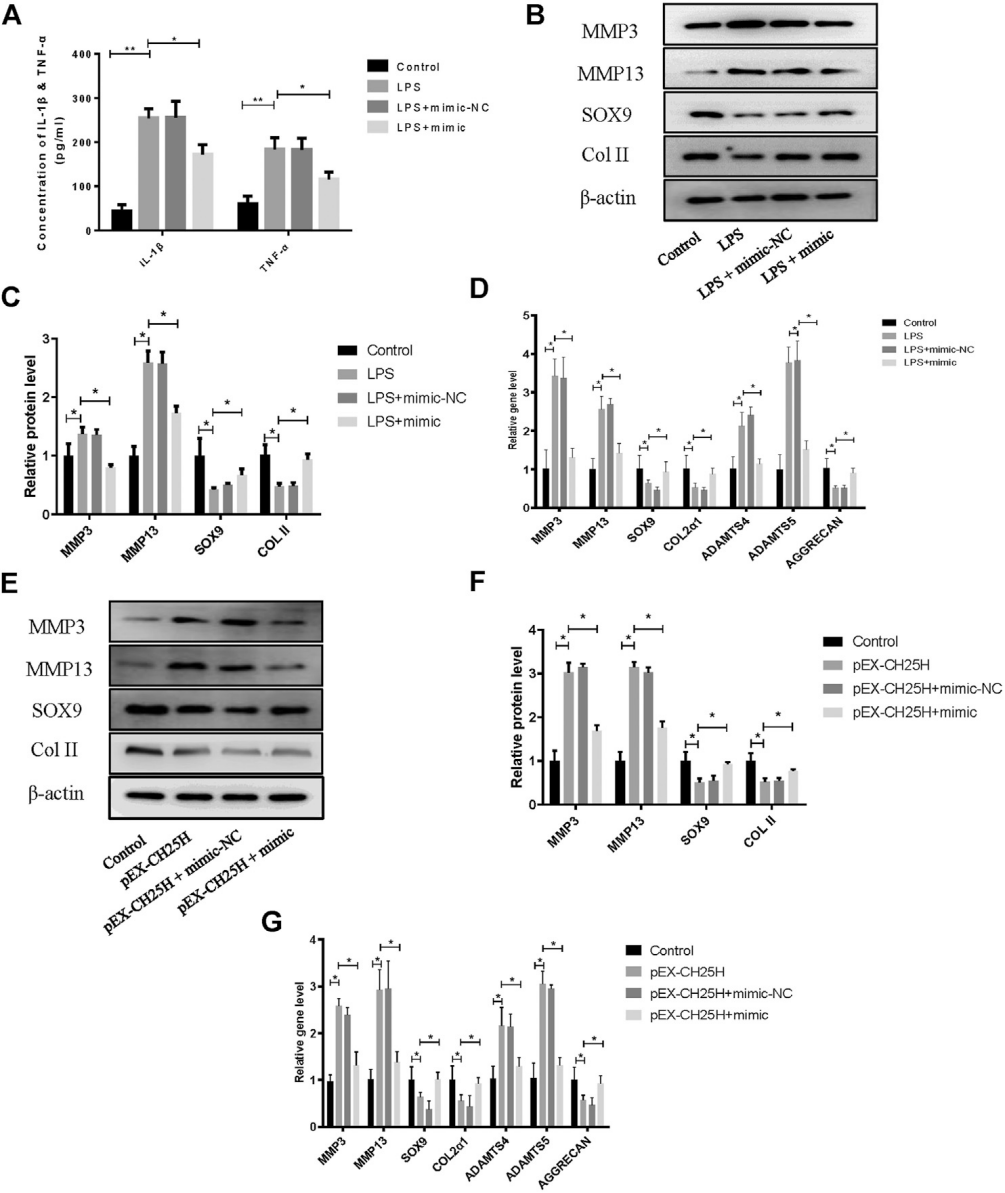
MiR-10a-3p regulates inflammation and extracellular matrix homeostasis in chondrocytes. **(A)** Concentrations of IL-1β and TNF-α in chondrocytes supernatant were detected by ELISA. **(B**,**C)** MMP3, MMP13, SOX9, and Collagen II expression in chondrocytes were analyzed via western blotting. The band intensity was quantified by normalizing to β-actin (*n* = 3). **(D)** MMP3, MMP13, SOX9, COL2α1, ADAMTS4, ADAMTS5, and Aggrecan expression in chondrocytes were analyzed via qRT-PCR. **(E, F)** MMP3, MMP13, SOX9, and Collagen II expression in chondrocytes were analyzed via western blotting. The band intensity was quantified by normalizing to β-actin (*n* = 3). **(G)** MMP3, MMP13, SOX9, COL2α1, ADAMTS4, ADAMTS5, and Aggrecan expression in chondrocytes were analyzed via qRT-PCR. Quantitative data were presented as mean ± SD. *p < 0.05, **p < 0.01.

To further investigate the role of miR-10a-3p in the progression of OA, we first transfected miR-10a-3p mimic into LPS-stimulated chondrocytes. The inflammatory responses are established to promote chondrocyte ECM degradation (Min et al., 2021), therefore, we first detected levels of representative inflammatory factors, IL-1β and TNF-α, in supernatant of chondrocytes by ELISA. The results showed that miR-10a-3p mimic suppressed the enlarged concentration of IL-1β and TNF-α induced by LPS stimulation (Figure 5A). As shown in Figures 5B,C, miR-10a-3p reduced the production of extracellular matrix (ECM) degradation related protein, MMP3 and MMP13. Also, miR-10a-3p enhanced the production of ECM synthesis related protein, SOX9 and Collagen II. In addition, ECM degradation and synthesis related gene, including MMP3, MMP13, Adamts4, Adamts5, SOX9, Col2α1, and Aggrecan were detected by qRT-PCR. As shown in Figure 5D, miR-10a-3p suppressed MMP3, MMP13, Adamts4, and Adamts5 expression and increased SOX9, Col2α1, and Aggrecan expression. Then, we co-transfected CH25H plasmids and miR-10a-3p mimic into primary chondrocytes. Our data also showed that overexpression of CH25H could enhanced the expression of MMP3 and MMP13 and reduced expression of SOX9 and Collagen II in both mRNA and protein level in chondrocytes. MiR-10a-3p mimic partially reversed these effects of CH25H overexpression (Figures 5E–G). Taken together, miR-10a-3p partially recovered ECM degradation of chondrocyte induced by CH25H overexpression.

### MiR-10a-3p Improves Cartilage Degeneration in KOA Model Rats

**FIGURE 6:**
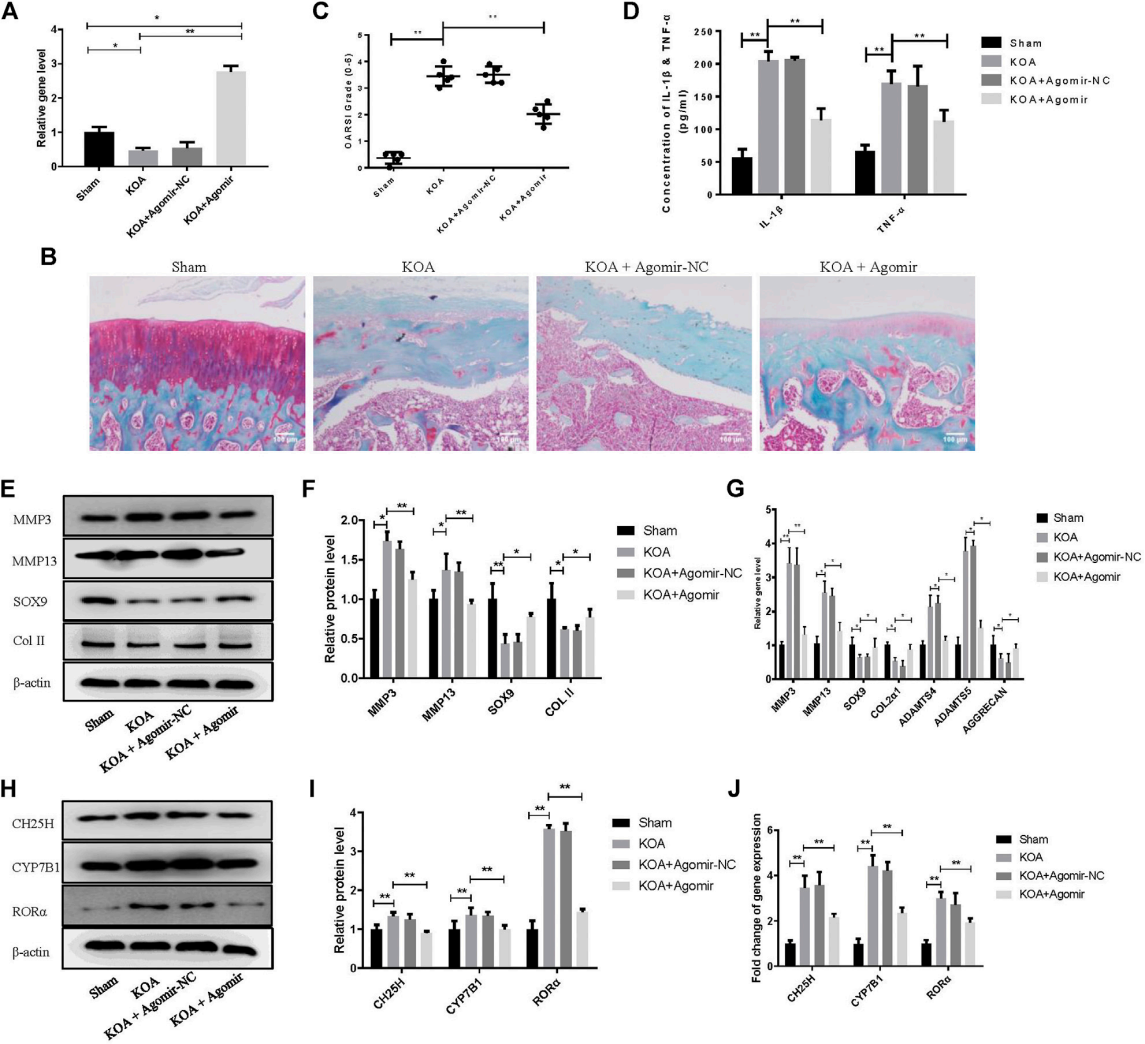
MiR-10a-3p improves cartilage degeneration in KOA model rats. **(A)** Detection of transfection efficiency of miR-10-3p agomir by qRT-PCR. **(B)** Saffron Red & Fast green staining of cartilage tissue, 100×, Scale bar = 100 μm. **(C)** OARSI scores of cartilage tissue in different groups (*n* = 5). **(D)** Concentration of IL-1β and TNF-α in serum were detected by ELISA. **(E**,**F)** MMP3, MMP13, SOX9, and Collagen II expression in cartilage tissue were analyzed via western blotting. The band intensity was quantified by normalizing to β-actin (*n* = 3). **(G)** MMP3, MMP13, SOX9, COL2α1, ADAMTS4, ADAMTS5, and Aggrecan expression in cartilage tissue were analyzed via qRT-PCR. Quantitative data were presented as mean ± SD. **(H**,**I)** CH25H, CYP7B1, and RORα expression in cartilage tissue were analyzed via western blotting. The band intensity was quantified by normalizing to β-actin (*n* = 3). **(J)** CH25H, CYP7B1, and RORα expression in cartilage tissue were analyzed via qRT-PCR. Quantitative data were presented as mean ± SD. *p < 0.05, **p < 0.01.

Model of knee OA rats was established to probe the role of miR-10a-3p in OA. Our data showed that the expression of miR-10a-3p was downregulated in KOA group and was upregulated by injection of miR-10a-3p agomir (Figure 6A). The safranine O and fast green staining of cartilage tissue indicated that miR-10a-3p agomir alleviated cartilage degeneration in OA rats which was also proved by OARSI’s scores (Figures 6B,C). Compared with KOA group, the OARSI’s scores were decreased in KOA + Agomir group. The mRNA expression of IL-1β and TNF-α were enhanced in KOA group compared with sham group. In addition, injection of miR-10a-3p agomir reversed the increase of IL-1β and TNF-α mRNA expression in KOA group (Figure 6D). As showed in Figures 6E–G, overexpression of miR-10-3p could neutralize the increase of MMP3 and MMP13 as well as the decrease of SOX9 and Collagen II in both protein and mRNA expression in KOA model rats. These findings suggest that miR-10-3p can suppress OA progression via targeting CH25H *in vivo*.

## Discussion

OA has become a global challenges for the elderly. The main pathological features are cartilage matrix degradation and synovium inflammation (Chen et al., 2021). While much has been learned recently regarding the pathogenesis of OA, the treatment outcome is also unsatisfactory due to the unclear pathogenesis. Recently, more and more researches suggest OA can be attributed to the metabolic imbalances (Kulkarni et al., 2021). Abnormal lipid profile exhibited in obesity, contributes to OA pathology (Klop et al., 2013; Abente et al., 2016). Generalized OA related serum hypercholesterolemia has indicated cholesterol as a systemic risk factor for it.

CH25H is a primary effector in controlling cholesterol metabolism and its dysregulation affects various diseases. For instance, CH25H, hydroxylates cholesterol to 25-HC, exerts promotive effects on the adipose tissue inflammation in obesity and diabetes (Russo et al., 2020). Furthermore, CH25H/CYP7B1/RORα axis exhibits regulatory effects on enhancing catabolic metabolism of chondrocytes (Choi et al., 2019) which is consistent with this study. However, specific upstream regulator of CH25H in chondrocytes remains unknown.

Recently, miRNAs, one of ncRNAs that contains 20–23 nucleotides, have been proved to play a pivotal role in the pathology of OA, especially in the cartilage matrix remodeling. For example, miRNA-140^−/−^ mice show severe cartilage damage and miR-140 transgenic mice manifest ameliorative antigen-induced arthritis by regulating cartilage homeostasis (Duan et al., 2020). MiR-10a-5p, miR-126, miR-181a-5, and miR-320a can induce chondrocyte apoptosis in a model of IL-1β-induced OA (Jiang et al., 2020). MiR-10a-5p has been reported that upregulated and induced apoptosis through repression of HOXA1 in chondrocytes (Ma et al., 2019). Therefore, we performed high-throughput miRNA sequence to seek for upstream regulator of CH25H, which is miR-10a-3p.

Previous research has suggested that miR-10a plays an important role in osteoarthritis. A recent study revealed that miR-10a acts as a downstream of TWIST and upstream of MAP3K7 in OA and this axis can ameliorate synovitis in OA fibroblast-like synoviocytes (Tu et al., 2020). On the basis of previous studies, we further explored the mechanism of miR-10a-3p on the progress of OA. We revealed that miR-10a-3p was downregulated in osteoarthritic cartilage and chondrocytes. Moreover, miR-10a-3p alleviates OA development by regulating chondrocyte cholesterol metabolism and inhibiting ECM degradation.

Regulation of miRNAs is a common phenomenon that occurs in various diseases, and aberrantly expressed miRNAs often participate in the pathogenesis of specific diseases, including OA. MiRNAs commonly exert their functions through targeting 3′UTR of their target genes. In current research, we found that miR-10a-3p exhibited binding sites on the 3′ UTR of CH25H and further confirmed the binding capability of miR-10a-3p and CH25H. Moreover, miR-10a-3p mimic administration can reverse CH25H-medicated effects on cholesterol metabolism and hydroxylation, proinflammatory cytokine production and ECM degradation both *in vitro* and *in vivo* were revealed by rescue assay.

**FIGURE 7:**
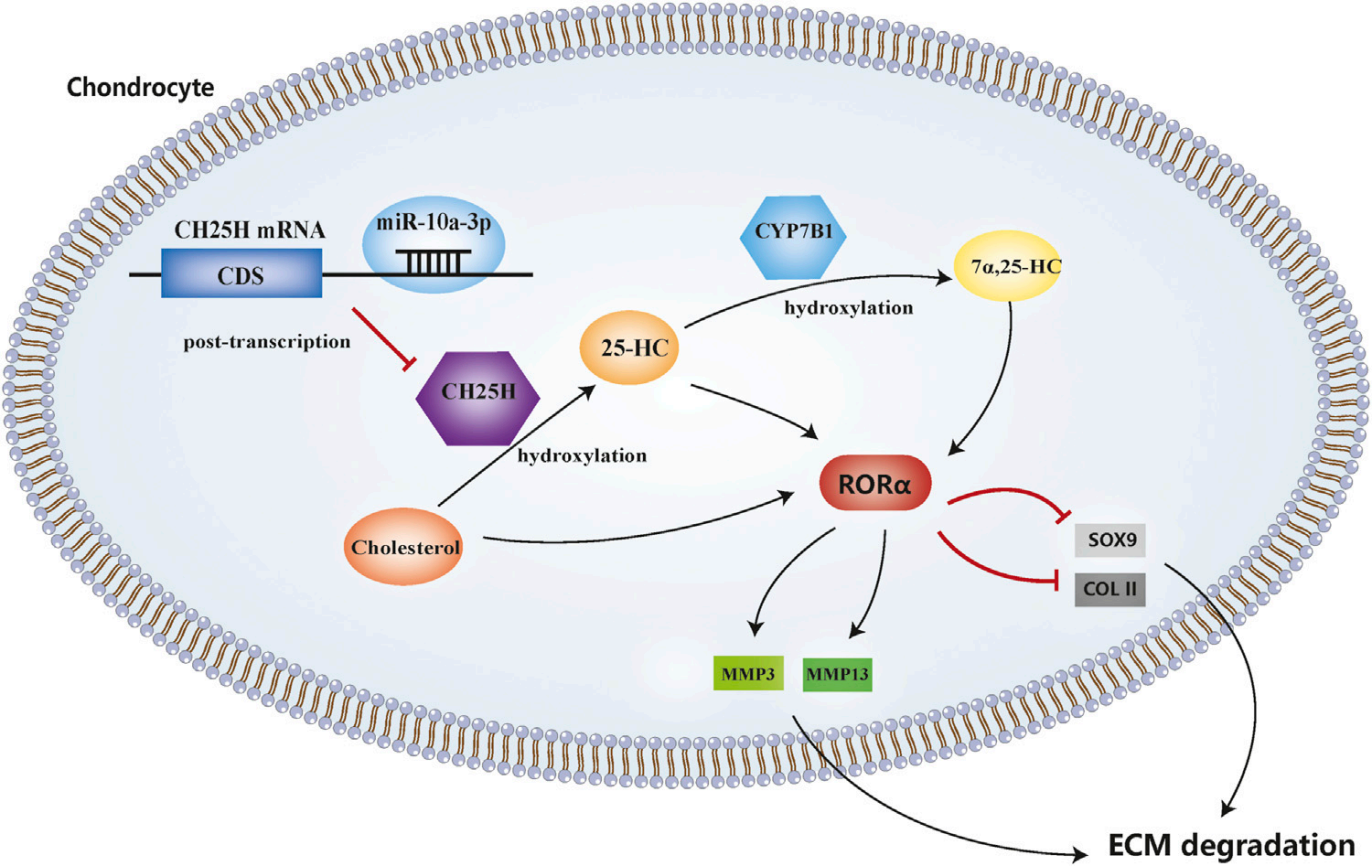
MicroRNA-10a-3p improves ECM degeneration by regulating CH25H-CYP7B1-RORα mediated cholesterol metabolism in osteoarthritic chondrocytes.

In this study, we demonstrated that miR-10a-3p can regulate chondrocyte cholesterol metabolism, including hydroxylation of cholesterol, by targeting CH25H mRNA and its downstream signaling CYP7B1 and RORα (Figure 7).

## Data Availability

The datasets presented in this study can be found in online repositories. The names of the repository/repositories and accession number(s) can be found in the article/Supplementary Material.
