# Supplementary material for: MicroRNA-10a-3p Improves Cartilage Degeneration by Regulating CH25H-CYP7B1-RORα Mediated Cholesterol Metabolism in Knee Osteoarthritis Rats
Source: Front Pharmacol. 2021 Jun 3;12:690181. doi: 10.3389/fphar.2021.690181 (PMC8209416; doi:10.3389/fphar.2021.690181)
Supplement: Supplementary file 1 [file Table1.DOCX]

Ch25h(NM_001025415-3utr(mir-10a-3p))

ACGCAAGAAAAATCAGAGAGATCCTCATAAAGGCCAAGAAGGGCGGAAAGATCGCCGTGTAATTCTAGAGTTATTTTTTCAGAAGTTAGTACATTTTTAAGTGATGAACACTGCTATAAAATCTAATGTGTTTCTGCAGCCCGACAAAGTAATTTATATAATGTTTATATATGAATTTAATTGTGGTCTTGATGTCAAATTCCAACTCGTCCCAGTGTCCTTGACTTCAGGACACAAGTTAGACATTCTCTAGAATGGTTTGTTTGTAGAACCAAGACTTTCCTATGTATGAAACTGCACTGGAACAGGGCTAATCACGCATGTTGATGGAAGACCTGATGTCTGACTCTTTAAAATAATCAAATTTGTTCCTGCTGGGCAGTGATCAAGATTATAGTATATTTTTTCTATTTTTGAGCAAAATGATATATTTATATTGAAAGAATTTTTATTCGTATTTTAAAAATAAAAAGAACATGAACTAAATCTAGAGTCGGGGCGGCCGGCCGCTTCGAGCAGACATGATAAGATACATTGATGAGTTTGGACAAACCACAACTAGAATGCAGTGAAAAAAATGCTTTATTTGTGAAATTTGTGATGCTATTGCTTTATTTGTAACCATTATAAGCTGCAATAAACAAGTTAACAACAACAATTGCATTCATTTTATGTTTCAGGTTC

Ch25h(NM_001025415-3utr(mir-10a-3p)-mut)

GCAAGAAAAATCAGAGAGATCCTCATAAAGGCCAAGAAGGGCGGAAAGATCGCCGTGTAATTCTAGAGTTATTTTTTCAGAAGTTAGTACATTTTTAAGTGATGAACACTGCTATAAAATCTAATGTGTTTCTGCAGCCCGACAAAGTAATTTATATAATGTTTATCGCGTCCGGGAATTGTGGTCTTGATGTCAAATTCCAACTCGTCCCAGTGTCCTTGACTTCAGGACACAAGTTAGACATTCTCTAGAATGGTTTGTTTGTAGAACCAAGACTTTCCTATGTATGAAACTGCACTGGAACAGGGCTAATCACGCATGTTGATGGAAGACCTGATGTCTGACTCTTTAAAATAATCAAATTTGTTCCTGCTGGGCAGTGATCAAGATTATAGTATATTTTTTCTATTTTTGAGCAAAATGATATATTTATATTGAAATCCGGGTTATTCGTATTTTAAAAATAAAAAGAACATGAACTAAATCTAGAGTCGGGGCGGCCGGCCGCTTCGAGCAGACATGATAAGATACATTGATGAGTTTGGACAAACCACAACTAGAATGCAGTGAAAAAAATGCTTTATTTGTGAAATTTGTGATGCTATTGCTTTATTTGTAACCATTATAAGCTGCAATAAACAAGTTAACAACAACAATTGCATTCATTTTATGTTTCAGGTTCAGGGGGAGGTG
